# Prediction of pedicled flap survival preoperatively by operating indocyanine green angiography at 1,450 nm wavelength: an animal model study

**DOI:** 10.3389/fmed.2024.1389384

**Published:** 2024-05-20

**Authors:** Chenchen Shi, Linxiumei Guo, Ruihu Song, Heng Xu, Yixin Zhang

**Affiliations:** ^1^Department of Plastic and Reconstructive Surgery, Shanghai Ninth People’s Hospital, Shanghai Jiao Tong University School of Medicine, Shanghai, China; ^2^Molecular Imaging Center, State Key Laboratory of Chemical Biology, Shanghai Institute of Materia Medica, Chinese Academy of Sciences, Shanghai, China

**Keywords:** indocyanine green angiography, near-infrared, pedicled flap, choke vessel, angiosome

## Abstract

**Background:**

Predicting flap viability benefits patients by reducing complications and guides flap design by reducing donor areas. Due to varying anatomy, obtaining individual vascular information preoperatively is fundamental for designing safe flaps. Although indocyanine green angiography (ICGA) is a conventional tool in intraoperative assessment and postoperative monitoring, it is rare in preoperative prediction.

**Methods:**

ICGA was performed on 20 male BALB/c mice under five wavelengths (900/1,000/1,100, /1,250/1,450 nm) to assess vascular resolution after ICG perfusion. A “mirrored-L” flap model with three angiosomes was established on another 20 male BALB/c mice, randomly divided into two equal groups. In Group A, a midline between angiosomes II and III was used as a border. In Group B, the points of the minimized choke vessel caliber marked according to the ICG signal at 1,450 nm wavelength (ICG_1450_) were connected. Necrotic area calculations, pathohistological testing, and statistical analysis were performed.

**Results:**

The vascular structure was clearly observed at 1,450 nm wavelength, while the 900 to 1,100 nm failed to depict vessel morphology. Necrosis was beyond the borderline in 60% of Group A. Conversely, 100% of Group B had necrosis distal to the borderline. The number of choke vessels between angiosomes II and III was positively correlated with the necrotic area (%). The pathohistological findings supported the gross observation and analysis.

**Conclusion:**

ICG_1450_ can delineate the vessel structure in vivo and predict the viability of pedicled skin flaps using the choke vessel as the border between angiosomes.

## 1 Introduction

Skin flaps are widely used in reconstructive surgery for soft tissue defects caused by trauma, burns, or oncologic resection (1–4). Nevertheless, skin necrosis is a common complication, occurring in 20–30% of pedicled flaps (5, 6). This complication leads to unexpected consequences, such as re-operation, infection, delayed healing, and additional costs (7). To decrease morbidity, accurately predicting flap is a critical step in skin flap transfer.

Reliable blood flow is indispensable for flap survival. Several adjuvant tools, such as indocyanine green angiography (ICGA), laser Doppler, and tissue oxygen saturation measurements, have been developed to assess flap perfusion (1, 8, 9). Koolen et al. (10) developed an oxygen-sensing paint-on bandage providing both wound protection and constant wound oxygenation assessment. While these tools are valuable in predicting necrotic areas to modify flaps by removing the part of the “dark area,” their main drawback is that they are applied intraoperatively or postoperatively (4, 5). Due to existing nonviable tissue and extra skin that has been harvested, intraoperative design serves as a “second guesser,” resulting in unnecessary damage in donor sites (4). To address this problem, preoperative prediction is a promising approach. Nevertheless, the principles and technology for accurate design remain key issues to be solved.

In 1987, Taylor and Palmer introduced the concept of “angiosome” to understand the anatomical structures of the skin tissue (11). Angiosomes are the fundamental component of tissue, consisting of a single source vessel supplying a three-dimensional block. Angiosomes are connected by either choke vessels with reduced vessel caliber or true anastomoses without change in caliber (11, 12). Other studies have indicated that choke vessels work as “border control” vessels. The source artery can supply its own and adjacent angiosomes, beyond which the blood supply is unreliable with the possibility of necrosis, and angiosomes are connected by choke vessels (13, 14). Therefore, preoperatively mapping the choke vessels connected to the angiosomes can theoretically predict flap viability and guide flap design. However, vascular anatomy varies among patients, and cadaver studies-driven anatomical knowledge fails to delineate these angiosomes individually (14). Therefore, a technology that can delineate angiosomes *in vivo* is required.

Current angiography methods can be generally divided into optical and non-optical methods such as computed tomographic angiography (CTA) and Doppler ultrasound. CTA is able to provide high-resolution vessel images, but its imaging modalities are not dynamic and real-time and has radiation. In addition, the CTA equipment is unportable. Although Doppler ultrasound is used to localize perforators, whose equipment is portable, it is unable to show the entire vascular network and its resolution is unsatisfactory. ICG is a Food and Drug Administration (FDA)-approved drug for angiography in microsurgery (15), with a maximum emission wavelength of approximately 800 nm. Thus, the clinically used fluorescence signal of ICG is approximately 800 nm, falling within the near-infrared I window (NIR-I, 700–900 nm) (16). This window offers exquisite sensitivity for noninvasive intra-and postoperative blood perfusion assessment to predict flap survival (8, 17); however, it is limited by scattering and autofluorescence, making it challenging to depict clear vessel structures. Recent studies have demonstrated that the ICGA skin blush location, size, or intensity obtained do not correlate with perforator location (18). As an advanced alternative, the NIR-II window (1,000–1,700 nm) can significantly reduce scattering and minimize autofluorescence, providing clearer vessel outlines (19). ICG has a non-negligible long emission tail that stretches into the NIR-II region (20, 21), offering higher vasculature resolution than the NIR-I window (20). Wu et al. (22) et al. mapped general vascular network of the anterolateral thigh flap with ICGA under 1,300 nm LP. Therefore, the ICG NIR-II fluorescent signal can delineate angiosomes *in vivo* in theory. Although other NIR-II dyes have been developed for flap angiography, such as quantum dots (23) and cyanine dyes (24), the clinical translation of drugs takes a long time.

In this study, we performed ICGA using a mouse model to validate the superiority of the NIR-II window over the NIR-I window. We established a three angiosomes flap model connected by caliber-reduced choke vessels to assess the preoperative reliability of the “angiosome principle” for predicting flap viability.

## 2 Materials and methods

### 2.1 Animals and flap design

Forty male BALB/c mice (6–8 weeks, 25–30 g) were used. All animal experiments were performed in accordance with the guidelines of the Ethics Committee of Shanghai Jiao Tong University.

**Figure 1:**
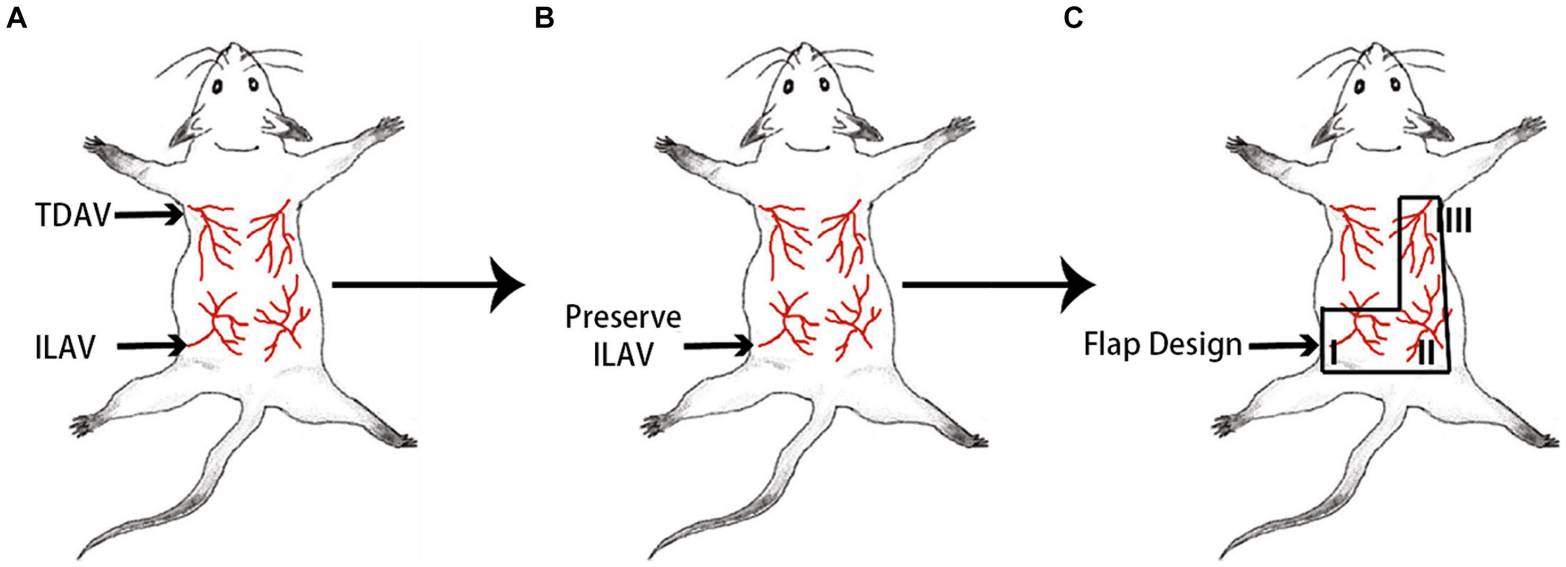
Design of a “mirrored-L” flap on the back area of the mouse. **(A)** The back area of the skin comprises four angiosomes. TDAV: thoracic dorsal artery/vein; ILAV: iliac lumbar artery/vein. **(B)** By preserving the ILAV on the left side, **(C)** a “mirrored-L” flap is designed to include three angiosomes. From the proximal to the distal, the three angiosomes are marked as I, II, and III, and the source vessels of angiosomes II and III are ligated.

A skin flap model was designed for the back area consisting of four angiosomes nourished by bilateral thoracic dorsal artery/vein and iliac lumbar artery/vein (ILAV) (Figure 1A) (25). A “mirrored-L” flap was designed, preserving only the ILAV on the left side while litigating the other two pedicles (Figure 1B). From the proximal to the distal part (based on the source vessels-ILAV), the three angiosomes were marked as I, II, and III (Figure 1C). The flap extended 1.5 cm laterally to each side of the spine (midline). The inferior border was drawn along the posterior superior iliac spine. Angiosome III had a superior border along the inferior angle of the scapula and a left border 0.5 cm lateral to the spine. Angiosome I had a superior border 0.5 cm inferior to the midline between the inferior angle of the scapula and posterior superior iliac spine.

### 2.2 ICGA

Mice were placed in the prone position and anesthetized with 2 L/min O_2_ gas mixed with 2% isoflurane. ICG (10 μg, 100 μL in ddH_2_O, Dandong Yichuang Pharmaceutical Co., Dandong, Liaoning, China) was injected intravenously before angiography. NIR fluorescence imaging was performed using an *in vivo* NIR imaging system (MARS; Artemis Intelligent Imaging, Shanghai, China). This NIR imaging system contained the NIRvana 640 detector, a light shield, and a movable working platform with a working distance from 400 mm to 1,000 mm. In this experiment, the focal length was approximately 330 mm. The room temperature must be maintained below 30°C and the water cooler kept the camera temperature at -80°C.

All experiments were conducted using 808 nm diode lasers (120 mW/cm^2^) as the excitation light source. Long-pass filters (LPs) at wavelengths of 900 nm, 1,000 nm, 1,100 nm, 1,250 nm, and 1,450 nm were used to obtain NIR fluorescence signals at different wavelengths. Each filter was mounted in a circular holder with a magnet which could be attached directly under the lens. The exposure times for the five filters were 3 ms, 5 ms, 10 ms, 100 ms, and 10 s, respectively, with the fluorescence intensity of the liver set as a standard (5,000–13,000 a.u.).

Twenty mice were used for ICGA to assess the differences between the five filters. Twenty seconds after injecting ICG, ensuring adequate drug perfusion in the vessels, each mouse was observed sequentially through all five filters. The caliber-reduced choke vessel around the midline was measured using Origin 2021 (OriginLab, Corp., Massachusetts, United States) to compare the imaging resolutions at different wavelengths. A cross-sectional analysis was performed across the vessel, and the full width at half-maximum was calculated using a Gaussian function as the vessel diameter.

### 2.3 Group division and skin flap model

Based on the different methods for delineating the border between angiosomes II and III, 20 mice were randomly divided into two groups (*n* = 10/group). Mice of the two groups were randomly selected from cages. No blinding was performed.

**Figure 2:**
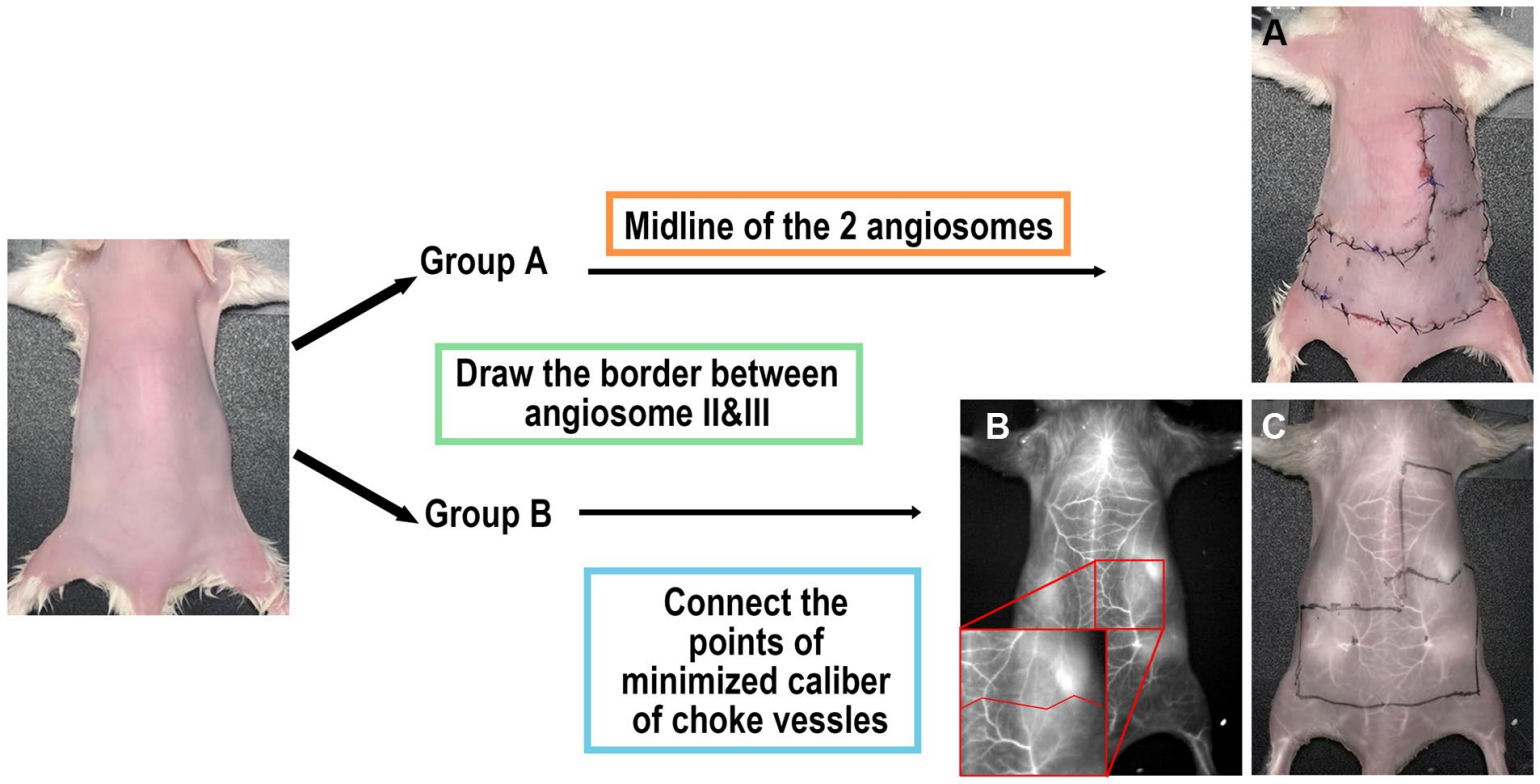
Scheme of study design. **(A)** Group A: surgery performed without imaging assistance. The midline of the two angiosomes was considered the border. **(B,C)** Group B: The operation was performed with ICGA assistance. The border was determined using images obtained under 1,450 nm LP.

Group A: The borderline was drawn along the midline between the inferior angle of the scapula and the posterior superior iliac spine (Figure 2A).

Group B: The borderline was drawn to connect the locations with minimized caliber at each vessel (Figure 2C). The number of choke vessels at the border was recorded at the same time by LG.

All mice were anesthetized by intraperitoneal injection of 10% Zoletil* 50 mixed with xylazine (0.5 mg/mL) (10 μL/g) and underwent hair removal in the back area. According to the flap design, the “mirrored-L” skin flap was harvested with the left ILAV as a pedicle while the other vessels were ligated. The flaps were then sutured back in place using 6–0 polydioxanone sutures. All imaging and surgeries were performed by CS.

In Group A, the border between angiosomes II and III was marked directly. In Group B, ICGA was initially performed to obtain a clear vascular anatomy under 1,450 nm wavelength (Figure 2B). Caliber-reduced choke vessels residing in the area between angiosomes II and III were identified to establish the locations of the minimized caliber. Finally, a border was marked to connect all these points. We predicted that necrosis of the flap would occur distal to the border.

### 2.4 Necrotic area assessment

Seven days postoperatively, the flaps were photographed under a bright light to assess the percentage of necrotic areas in angiosome III. Each picture was imported into Image J version 1.53c (National Institutes of Health, Bethesda, MD, United States). The percentage of the necrotic area was calculated using the following formula: necrotic area (%) = necrotic area/area of angiosome III × 100.

### 2.5 Histopathology

Harvested flaps were fixed using 4% polyformaldehyde overnight, embedded in paraffin, and sectioned with a thickness of 5 μm for pathohistological slide mounting. The slides were stained with hematoxylin and eosin (H&E) using a standard protocol.

### 2.6 Statistical analysis

GraphPad Prism version 9.5.0 (GraphPad Software, Inc., San Diego, United States) was used for one-way ANOVA (and parametric tests) to compare full width at half maximum. Correlation analysis was performed using the Pearson correlation coefficient, *r*, between the number of choke vessels and the necrotic area at a 95% confidence level. A value of *p* < 0.05 was considered statistically significant. A value of |*r*| > 0.3/0.5/0.8 was considered to be low/moderate/high correlated, and *p* < 0.05 was considered a significant linear correlation between the two. Results are expressed as means (±SEM).

## 3 Results

### 3.1 Comparing the imaging resolution under different NIR wavelengths

**Figure 3:**
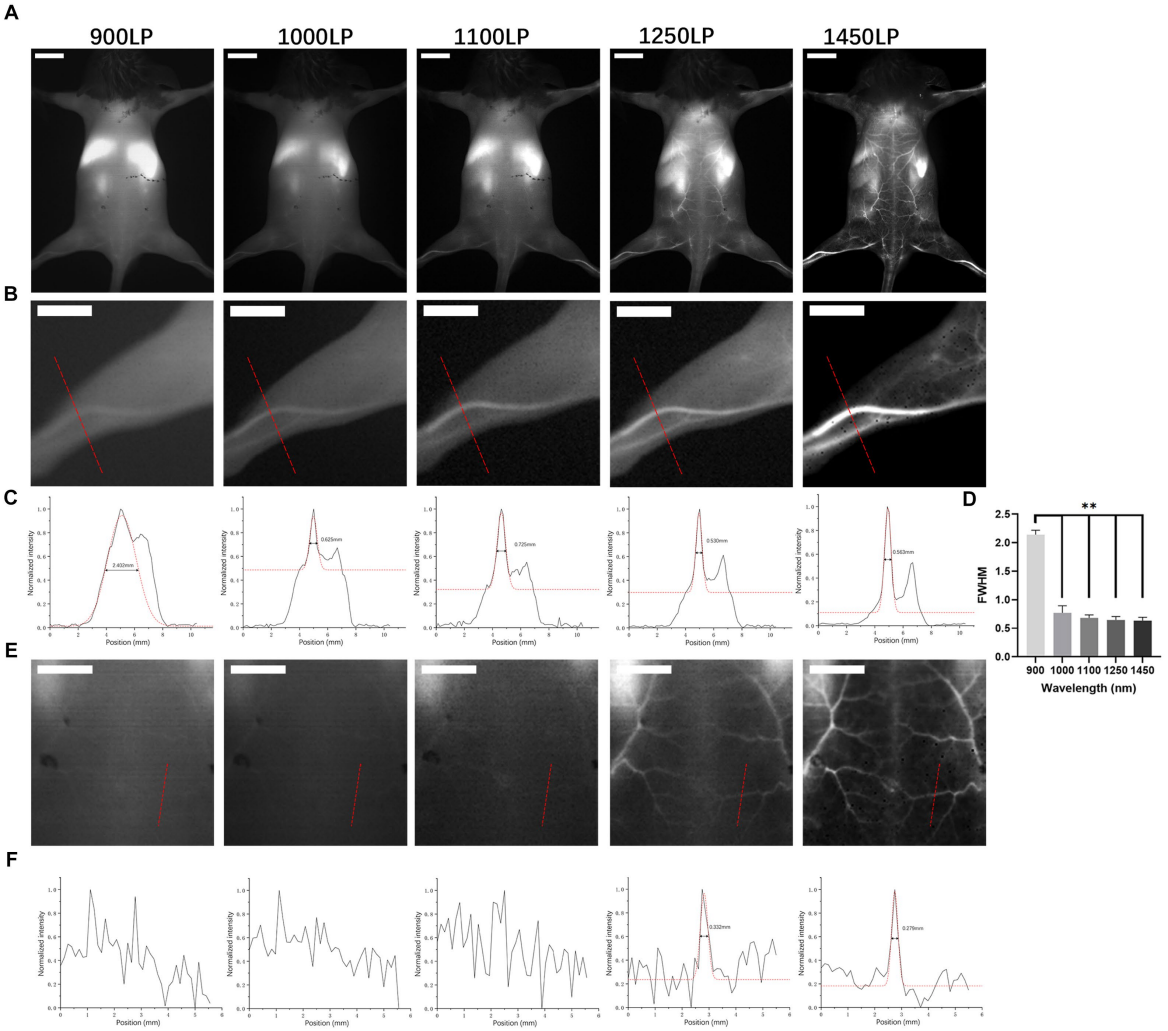
*In vivo* NIR images in BALB/c mice. **(A)** NIR fluorescence images of BALB/c mice after ICG solution injection under different long-pass filters (900 nm, 1,000 nm, 1,100 nm, 1,250 nm, and 1,450 nm). Scale bar = 1 cm. **(B,E)** Partial enlarged back part of A. Scale bar = 0.5 cm. **(C,F)** Cross-sectional analysis of the fluorescence intensity of the red line in **(B,E)**. **p < 0.01. **(D)** Full width at half maximum of Gaussian fits of hind limb vessels obtained under different long-pass filters mentioned above (*n* = 10 per group).

As illustrated in Figure 3, each mouse underwent ICGA to obtain the vascular anatomy using 900 nm, 1,000 nm, 1,100 nm, 1,250 nm, and 1,450 nm (Figure 3A) filters separately. The left hind legs (Figure 3B) and areas between angiosomes I and II (Figure 3E) were magnified to compare the imaging resolution. The 900 nm wavelength presented a fuzzy vessel structure, and the longer the wavelengths, the better the resolution achieved. The 1,450 nm wavelength achieved a clear vascular structure. We performed a cross-sectional analysis across the distal femoral vessel (Figure 3C) and a chosen branch of the cutaneous perforator (Figure 3F) and used a Gaussian function to measure the vessel caliber. For distal femoral vessels, relatively large vessels, we were able to identify a peak in profiles of all wavelengths and fit it into a Gaussian function. However, widths extracted under 900 nm were two to three folds broader than those under other LPs (*p* < 0.01), and there was no significant difference among 1,000 nm to 1,450 nm (Figure 3D), suggesting that it is feasible to locate a large (>500 μm) vessel but not reliable to calculate vessels diameter in NIR I window. As images of the branch vessels showed obscure vascular anatomy (Figure 3E) under 900–1,100 nm, 900–1,100 nm failed to present the peak of the fluorescence signal for measurement. In contrast, we identified one sharp peak in profiles of both 1,250 nm and 1,450 nm with a calculated diameter of 0.332 mm and 0.279 mm, respectively. Therefore, compared with 900 nm in the NIR-I window and 1,000–1,250 nm in the NIR-II window, the 1,450 nm wavelength displayed a clear vascular anatomy.

### 3.2 Flap viability

**Figure 4:**
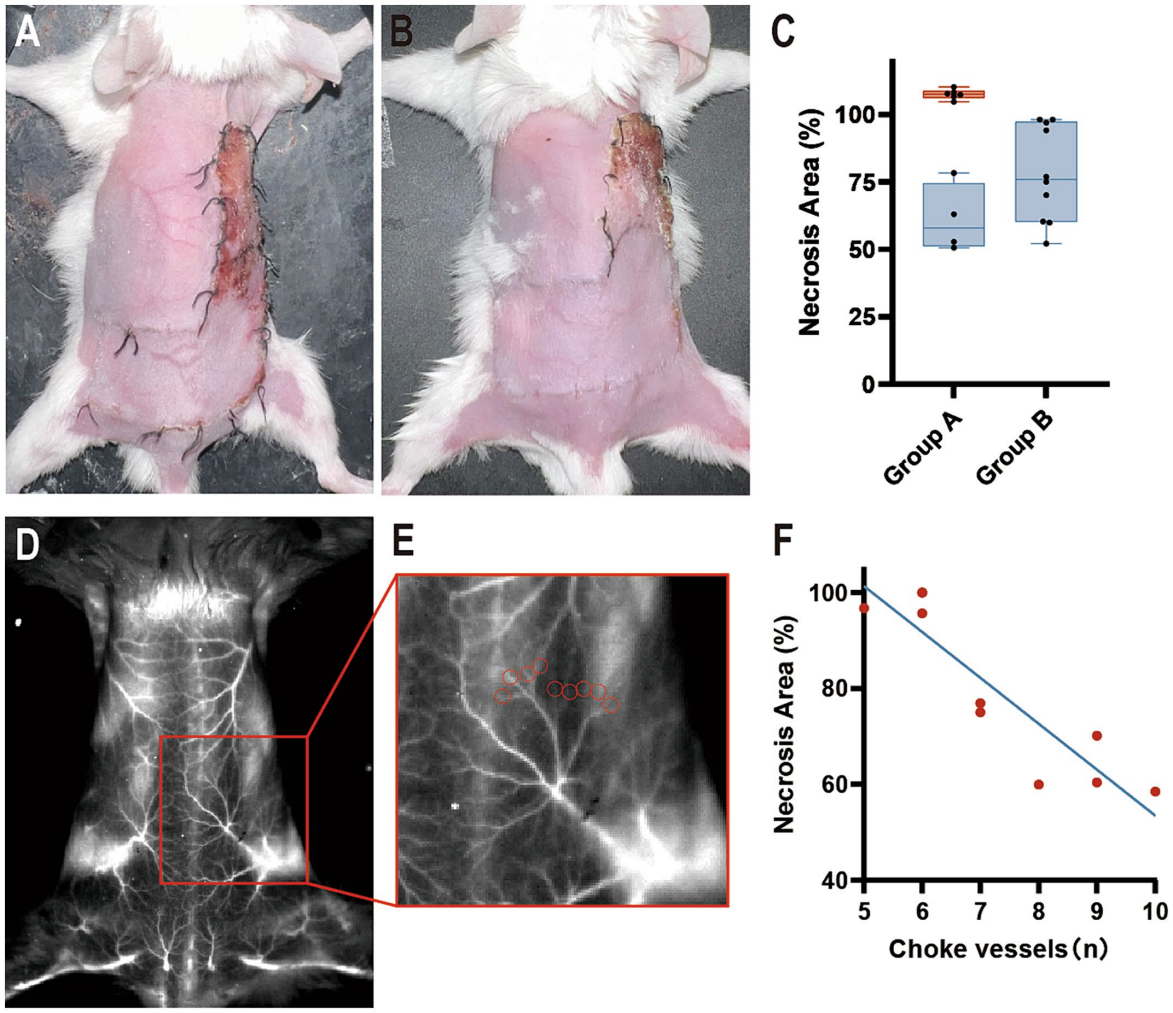
Flap survival at postoperative day 7. **(A)** Group A: Part of the necrosis was located outside angiosome III. **(B)** Group B: The necrotic area was located within angiosome III. **(C)** The percentage is calculated as a ratio of the necrotic area in angiosome III. Blue boxes indicate the necrotic area (%) <100, and the red box indicates the necrotic area (%) ≥100 (*n* = 10 per group). **(D,E)** ICGA_1450_ of **(B)** and its partially enlarged back part. Red circles indicate choke vessels between angiosomes II and III. **(F)** Correlation analysis with Pearson *r* between the number of choke vessels and the necrotic area.

In Group A, the “mirrored-L” skin flap model was set up directly, and the border between angiosomes II and III was drawn transversely as the midline of the two angiosomes. On postoperative day 7, the necrotic area was either larger or smaller than the drawn area of angiosome III (Figure 4A). The necrotic area (%) was 89.00 ± 25.02 (71.11–106.9%).

In Group B, mice underwent ICGA to delineate the border between angiosomes II and III by connecting the points of the minimized caliber of the choke vessels. The flap model was established for observation on postoperative day 7. In all mice, the necrotic area was completely included within the drawing area of angiosome III (Figure 4B). The necrotic area (%) was 78.22 ± 17.68 (65.57–90.87%).

Necrosis was beyond the borderline in 60% of Group A, while 100% of Group B had necrosis distal to the borderline (Figure 4C). With the help of ICGA_1450_, we were able to calculate the number of choke vessels between angiosomes II and III (Figures 4D,E). The r value was-0.9041, which implied a strong negative correlation between the number of vessels and necrotic area, and *p* < 0.05 suggested a linear relationship (Figure 4F). In addition, partial flap necrosis was observed in the remote area of angiosome II.

### 3.3 Histopathological findings

**Figure 5:**
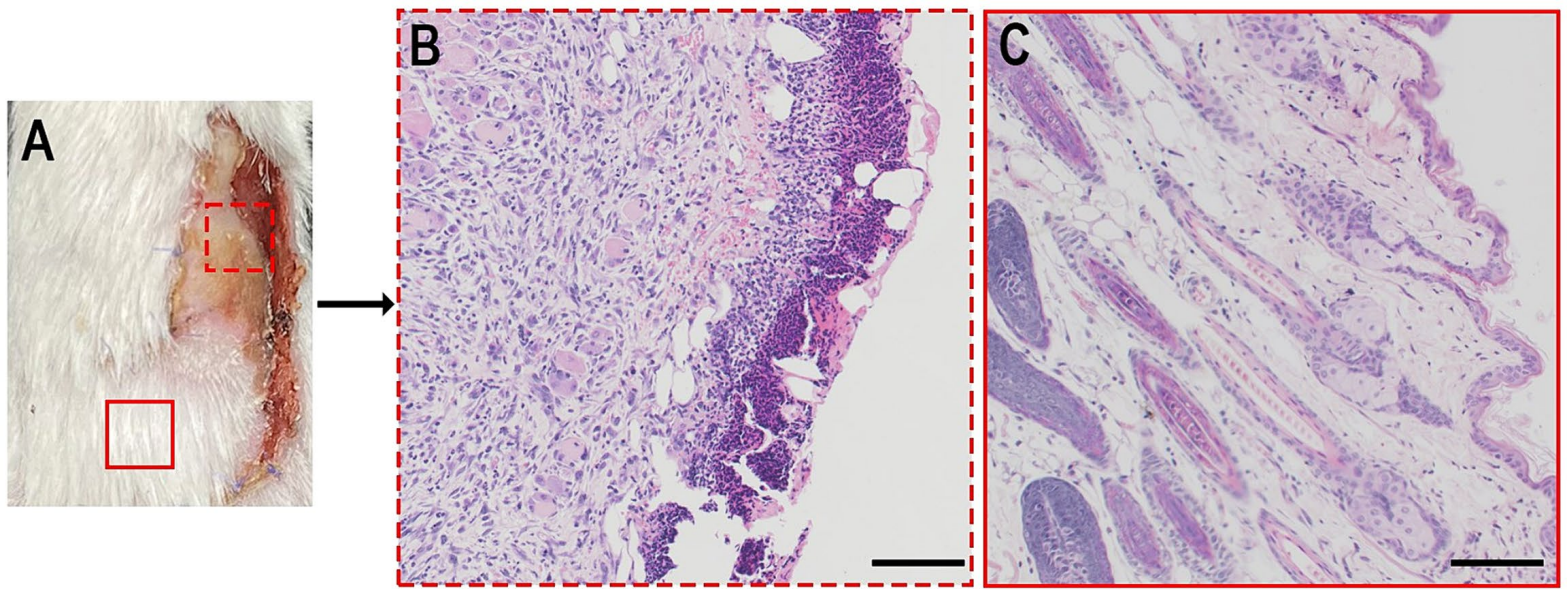
**(A)** Bright photo of a dorsal flap 7 days postoperatively. **(B,C)** H&E analysis of a certain zone of a. Scale bar = 100 μm.

H&E staining was performed to validate flap viability in gross view (Figure 5A). Tissues harvested from the necrotic area had a necrotic crust and many mononuclear cells infiltrating into the tissue without a clear skin structure (Figure 5B). Surviving tissues exhibited few mononuclear cell infiltrations and clear skin structures (Figure 5C).

## 4 Discussion

The design of safe flaps benefits the patients by reducing the rates of complications, such as partial necrosis (4, 14). Several anatomical and animal studies have offered several principles for flap design; however, varied vascular anatomy among individuals raises uncertainty in clinical practice (14, 26–28). In 2011, Taylor et al. emphasized that obtaining vascular anatomy information preoperatively is fundamental for the design of safe flaps (14). Considering this concept, we explored a feasible approach.

NIR laser-assisted ICGA evaluates flap viability by perfusing the vessels (1). Theoretically, this technique can present individual vascular anatomy information. However, clinical practice has revealed that only areas of different fluorescence signal intensities are observed (29, 30). This situation raises two questions: (1) what factors contribute to the differences between theory and practice, and (2) can ICGA be improved for observing vascular anatomy clearly?

The window for observing the fluorescence signals in ICGA is significantly correlated with vessel resolution. Clinically approved ICGA devices detect signals in the 800–860 nm range, falling within the NIR-I window (21). This window is hindered by limited tissue penetration and considerable fluorescence scattering (31), which might cause unclear vascular structure in practice. A longer fluorescence wavelength leads to deeper tissue penetration, higher signal-to-background ratio, lower signal scattering, and lesser autofluorescence (19). ICG possesses emission wavelengths ranging from 800 nm to 1,500 nm (21). Utilizing the 1,000–1,500 nm wavelength of ICG, which is within the NIR-II window, has demonstrated significantly higher resolution than the NIR-I window. In delineating brain vasculature, ICG signals above 1,300 nm increase the vessel resolution by approximately 1.4 times compared to the traditional NIR-I region (20). Similar results have been reported in ICGA studies of mouse hindlimb vessels (19). Therefore, shifting the fluorescence signal imaging window from NIR-I to NIR-II can provide a clearer observation of the vascular anatomy.

To assess the feasibility of this hypothesis, the back area of each mouse was used for ICGA. From the NIR-I to the NIR-II window, the filters with wavelengths of 900/1,000/1,100/1,250/1,450 nm demonstrated that a longer wavelength could achieve clearer vessel resolution, including vessel distribution and caliber. This test validates the theoretical expectation that imaging ICG in the NIR-II window, especially at the 1,450 nm wavelength, can distinctly delineate vascular anatomy. Thus, this method can be used to obtain vascular anatomical structure preoperatively for flap design.

The back area of the mouse could be divided into four angiosomes under the 1,450 nm wavelength, which aligns with *in vitro* observation in the literature (25). A single-vessel perfused three-angiosome skin flap model was established to observe flap viability. According to the angiosome theory, caliber-reduced choke vessels act as “border control” to define each angiosome (13, 14). This principle or simply drawing a border at the midline of the two angiosomes results in different outcomes. The previous method completely encompasses the necrotic area of angiosome III, validating both the angiosome theory and ICGA in the NIR-II window. In contrast, the necrotic area of the latter method was either included within or beyond the border. Therefore, the accuracy of the latter method was too poor to predict flap viability. The choke vessel “border” varies among mice, ranging approximately 1 cm wide in Group B, and has an irregular shape. To validate the gross observations further, a histopathological test was performed, supporting the reliability of this analysis. Therefore, by defining caliber-reduced vessels as choke vessels for mapping angiosomes, ICG_1450_ can assist in the individual design of skin flaps preoperatively based on the angiosome theory.

Based on the results and analysis of this study, the technique of ICG_1450_ addresses two questions raised by the current clinically used ICGA: (1) the poor resolution of the NIR-I window restricts ICGA from presenting clear vascular anatomy, and (2) shifting signal observation to the NIR-II window, especially with ICG_1450_, can improve the resolution. Ultimately, ICG_1450_ can delineate angiosomes *in vivo* to design flap preoperatively. In clinical practice, when the subject is transferred to human, we are able to use ICG_1450_ to delineate the boundaries of angiosomes. This ensures that the flap is harvested within the boundaries, thereby avoiding the risk of postoperative necrosis.

Although ICG is a FDA approved drug, there are some challenges in the clinical translation of ICG_1450_. Patients who are allergic to iodine and ICG and have renal dysfunction are not eligible for ICG_1450_. Given the short half-life of ICG, inexperienced surgeons have to inject ICG repeatedly to obtain vascular anatomy information, which increases their risk of adverse effects. The introduction of NIR-II imaging system involves medical economics, A comparison of its price and the medical expenditure saved by reduced incidence of flap necrosis needs to be considered.

This study had some limitations. First, we defined vessel sections with minimized caliber as the borders between angiosomes. In Group B, some necrosis appeared only in a small portion of the distal end, while others occupied almost the entire angiosome III, and we hypothesized that the number of choke vessels contributed partly, which was verified by correlation analysis. A greater number of choke vessels led to a smaller area of distal necrosis, implying an extension of tissue blood perfusion. There are still other factors such as vessel diameter, operation time, etc. that may affect flap survival, so the statistical analysis is limited. While partial necrosis was observed in the remote area of angiosome II, these differences in flap viability were located in the caliber-reduced vessel sections. Therefore, defining and drawing the border between the two angiosomes, for instance, based on the minimized caliber or a specific caliber of choke vessels, directly contradicts the flap survival area. However, further investigation is required.

Second, mouse skin is thinner than human skin, which may achieve a lower resolution than that in the present study. ICG_1450_ requires 5–10 s of exposure to identify the signals, and human breathing movements may create fuzzy images. Nevertheless, Wu et al. (22, 32) utilized a 1,300 nm LP filter to visualize ICGA in pig pedicled flaps and human anterolateral thigh flap, which roughly distinguished vascular networks. Therefore, large animal studies or clinical trials are necessary to validate the ICG_1450_ technique and they are also necessary in clinical translation. It is important to note that patients who are allergic to iodine and ICG and have renal dysfunction are not eligible for ICG_1450_.

Third, the longest filter in our imaging system was set at 1,450 nm. Therefore, the filters should be updated to determine whether a longer filter can obtain clearer imaging.

Fourth, the camera view of the current device is inadequate for observing large flaps, such as deep inferior epigastric flaps. Therefore, the device should be advanced to meet clinical requirements.

Finally, this study only clarified the feasibility of the ICG_1450_ technique for designing skin flaps. Whether this technique is suitable for the design of muscular bone flaps requires further investigation.

Although ICG is an FDA approved drug, there are some challenges in the clinical translation of ICG_1450_. Given the short half-life of ICG, surgeries need to be trained to avoid repeated injection of ICG, which increases the risk of adverse effects. The introduction of NIR-II imaging system involves medical economics, A comparison of the cost of equipment purchase and the medical expenditure saved by reduced incidence of flap necrosis needs to be considered. Multi-phase clinical trials to verify the feasibility and necessity of ICG_1450_ are necessary steps to receive regulatory approval, which may take 18 to 48 months.

## 5 Conclusion

To design safe skin flaps, obtaining clear information about the vascular anatomy preoperatively is fundamental. This is the first study to demonstrate that the ICG_1450_ technique could map vascular structures in detail, aiding in the prediction of distal necrosis and the design of skin flaps preoperatively based on the angiosome theory. As ICG is a widely used FDA-approved drug and ICG_1450_ can be achieved by changing the camera parameters, this technique has a strong translational value to advance microsurgery.

Nevertheless, this was solely an animal study and has several limitations. The emerging questions raised by these findings require further basic studies and clinical trials to provide conclusive answers.

## Data availability statement

The original contributions presented in the study are included in the article/supplementary material, further inquiries can be directed to the corresponding author.

## Ethics statement

The animal study was approved by Ethics Committee of Shanghai Jiao Tong University. The study was conducted in accordance with the local legislation and institutional requirements.

## Author contributions

CS: Writing – original draft, Writing – review & editing. LG: Writing – original draft, Writing – review & editing. RS: Methodology, Writing – review & editing. HX: Formal analysis, Supervision, Writing – review & editing. YZ: Formal analysis, Funding acquisition, Supervision, Validation, Writing – review & editing.
